# No evidence for increased cell entry or antibody evasion by Delta sublineage AY.4.2

**DOI:** 10.1038/s41423-021-00811-8

**Published:** 2022-01-05

**Authors:** Prerna Arora, Amy Kempf, Inga Nehlmeier, Luise Graichen, Martin S. Winkler, Martin Lier, Sebastian Schulz, Hans-Martin Jäck, Stefan Pöhlmann, Markus Hoffmann

**Affiliations:** 1 Infection Biology Unit, German Primate Center, Kellnerweg 4, 37077 Göttingen, Germany; 2 Faculty of Biology and Psychology, Georg-August-University Göttingen, Wilhelmsplatz 1, 37073 Göttingen, Germany; 3 Department of Anesthesiology, University of Göttingen Medical Center, Georg-August University Göttingen, Robert-Koch-Straße 40, 37075 Göttingen, Germany; 4 Division of Molecular Immunology, Department of Internal Medicine 3, Friedrich-Alexander University Erlangen-Nürnberg, Glückstraße 6, 91054 Erlangen, Germany

**Keywords:** SARS-CoV-2, AY.4.2, Delta, Host cell entry, Antibody evasion, Cell biology, Immunology

## Abstract

Since the beginning of the COVID-19 pandemic, multiple SARS-CoV-2 variants have emerged. While some variants spread only locally, others, referred to as variants of concern, disseminated globally and became drivers of the pandemic. All SARS-CoV-2 variants harbor mutations relative to the virus circulating early in the pandemic, and mutations in the viral spike (S) protein are considered of particular relevance since the S protein mediates host cell entry and constitutes the key target of the neutralizing antibody response. As a consequence, mutations in the S protein may increase SARS-CoV-2 infectivity and enable its evasion of neutralizing antibodies. Furthermore, mutations in the S protein can modulate viral transmissibility and pathogenicity.

The Delta variant, B.1.617.2, is currently the main driver of the pandemic. The success of the Delta variant may be attributable to multiple factors, including increased host cell entry efficiency and improved evasion of neutralizing antibodies [1, 2,]. Moreover, several Delta sublineages that harbor additional mutations in the S protein have branched off from the parental B.1.617.2 lineage, and their capacity to spread and cause disease is incompletely understood.

Many European countries are currently experiencing a surge in SARS-CoV-2 infections that could push health systems to their limits. The SARS-CoV-2 lineage AY.4.2, which represents a sublineage of the Delta variant (B.1.617.2 lineage), is currently expanding in the UK [3] (Fig. 1a, b), where it is responsible for 2.1–19.4% of new cases [4]. However, it is currently unknown whether the AY.4.2 variant differs from the parental virus B.1.617.2 in terms of its infectivity and sensitivity to antibody-mediated neutralization.

**Fig. 1:**
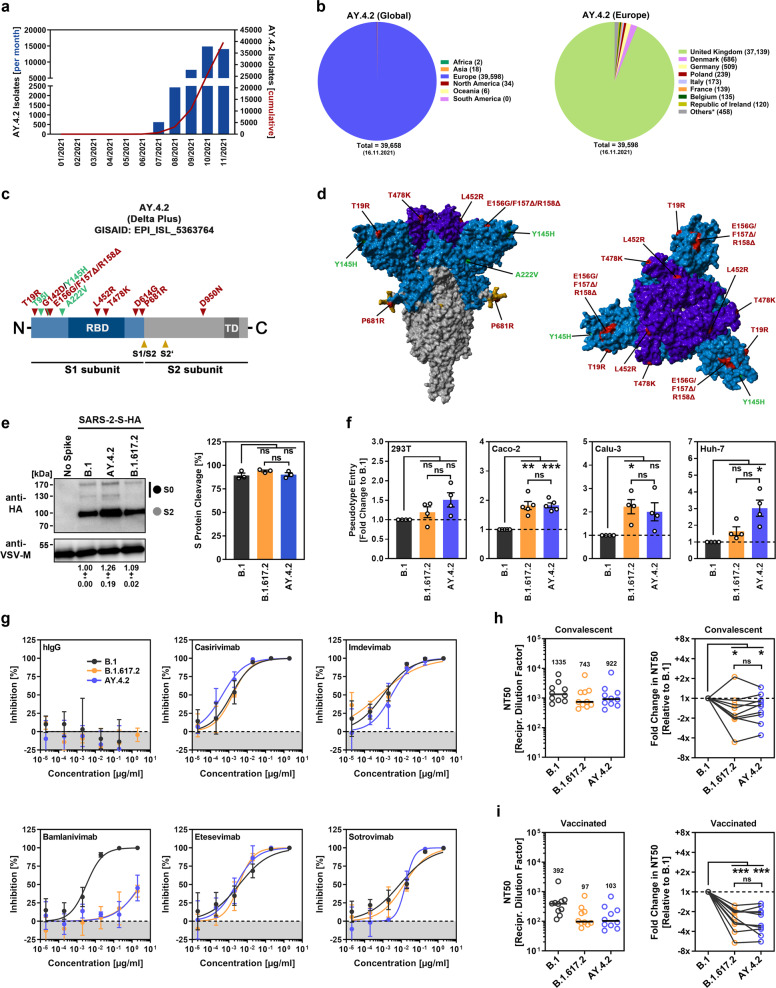
Host cell entry and antibody evasion by the spike protein of SARS-CoV-2 AY.4.2. **a** Monthly and cumulative numbers of globally reported AY.4.2 isolates. **b** Distribution of reported AY.4.2 isolates at the global (left) and European (right) levels. Numerical values in brackets indicate the number of isolates per country (* = twenty countries with < 100 isolates). **c** Schematic illustration of the SARS-CoV-2 spike protein in which the locations of functional domains (RBD, receptor binding domain; TD, transmembrane domain) and cleavage sites (S1/S2 and S2’) are highlighted. Mutations found in the spike protein of B.1.617.2 (Delta variant, EPI_ISL_1921353) are highlighted in red, while the additional mutations found in the Delta sublineage AY.4.2 (EPI_ISL_5633764) are highlighted in green. **d** Location of the amino acid changes in the context of the trimeric spike protein. **e** Pseudotyped particles bearing the indicated S proteins (equipped with a C-terminal HA epitope tag) were subjected to immunoblot analysis to analyze S protein incorporation and cleavage. S proteins and VSV-M (loading control) were detected using anti-HA and anti-VSV-M antibodies, respectively, in combination with a peroxidase-conjugated anti-mouse secondary antibody. The results from a single experiment are presented, and the results were confirmed in two additional experiments. For quantification of S protein incorporation, in all experiments, the S protein signals were first normalized against the corresponding VSV-M signals, and further incorporation of B.1 S protein was set as 1 (data represent the mean ± standard deviation, SD). For quantification of S protein cleavage, total S protein signals (bands representing unprocessed [S0] and processed [S2] S protein) for each S protein were set as 100%, and the respective proportions of S0 and S2 were calculated. Statistical significance was assessed by two-tailed Student’s *t* test with Welch’s correction; *p* > 0.05, not significant [ns]). **f** Four different human cell lines were inoculated with pseudotyped particles bearing the indicated spike proteins. At 16–18 h postinoculation, particle entry efficiency was analyzed by measuring the activity of virus-encoded luciferase in cell lysates. Presented are the average (mean) data from 4–5 independent experiments (each performed with four technical replicates) for which particle entry driven by the B.1 spike protein was set as 1. Error bars indicate the standard error of the mean. Statistical significance was assessed by two-tailed Student’s t test with Welch’s correction (*p* > 0.05, ns; *p* ≤ 0.05, *; *p* ≤ 0.01, **; *p* ≤ 0.001, ***; please see also Supplemental information, Fig. S1a). **g** Pseudotyped vectors bearing the indicated spike proteins were incubated (30 min, 37 °C) in the presence of different concentrations of monoclonal antibody or medium alone (control) before being added to Vero cells. Vector entry efficiency was analyzed at 16–18 h postinoculation and normalized against the respective control (set as 0% inhibition). Presented are the average (mean) data for a single experiment (with four technical replicates). The data were confirmed in a separate experiment. Error bars indicate the SD. Curves were calculated using a nonlinear regression model (variable slope). **h** Pseudotyped vectors bearing the indicated spike proteins were incubated (30 min, 37 °C) in the presence of different dilutions of convalescent plasma or only medium (control) before being added to Vero cells. Vector entry efficiency was analyzed at 16–18 h postinoculation and normalized to the respective control (set as 0% inhibition, please see Supplemental information, Fig. S1b for individual data). Furthermore, the plasma dilution that causes a reduction of 50% in vector entry (neutralizing titer 50, NT50) was calculated. Presented are the combined data for 10 convalescent plasma (each analyzed in four technical replicates). Black lines and numerical values indicate the median NT50. In addition, the data were normalized to reflect the relative change in neutralization sensitivity with the neutralization of B.1 spike serving as reference (set as 1, identical plasma are connected by lines). Statistical significance was assessed by Kruskal–Wallis analysis with Dunn’s multiple comparison test (*p* > 0.05, ns; *p* ≤ 0.05, *; *p* ≤ 0.001, ***). **i** The experiment was performed as described in (**h**), but serum from vaccinated individuals (BNT162b2/BNT162b2, *n* = 10) was analyzed (please see Supplemental information, Fig. S1c for individual data)

The S protein of AY.4.2 harbors the characteristic mutations of B.1.617.2 (Fig. 1c, d), including mutations L452R and T478K, which are located in the receptor binding domain (RBD), the portion of the S protein that directly engages the cellular receptor ACE2. These mutations have been shown to reduce the effectiveness of therapeutic antibodies and, together with mutations found in an antigenic supersite [5] within the N-terminal domain (NTD; G142D, E156D, F157Δ, R158Δ), likely enable the S protein to evade neutralizing antibodies elicited upon infection or vaccination [1]. Furthermore, the AY.4.2 S protein harbors the mutation P681R, which has been shown to augment S protein-driven cell–cell fusion, a process that is believed to contribute to coronavirus disease 2019 (COVID-19) pathogenesis [6, 7]. In comparison to B.1.617.2, the AY.4.2 S protein contains three additional mutations in the NTD (T95I, Y145H, and A222V), one of which (Y145H) is located in the antigenic supersite.

We first analyzed the AY.4.2 S protein for its ability to drive entry into target cells using a vesicular stomatitis virus (VSV) pseudotyped with S protein, which is a well-established surrogate model for SARS-CoV-2 cell entry [8]. AY.4.2 S protein was robustly incorporated into VSV particles and efficiently cleaved (Fig. 1e). For comparison, we evaluated the S proteins of B.1.617.2, Delta variant, and B.1, a lineage that circulated in the early phase of the pandemic. Compared to the S protein of B.1, both AY.4.2 and B.1.617.2 S proteins enabled augmented (~2-fold) entry into the human lung- and colon-derived cell lines Calu-3 and Caco-2, respectively, while entry into the kidney-derived 293T cell line was equal to that of B.1 (Fig. 1f). While the results for B.1.617.2 were in line with published data [1], no differences in entry efficiency were observed between AY.4.2 and B.1.617.2 S proteins, with the exception of a moderately more efficient (~2-fold, not statistically significant) entry into the human liver Huh-7 cell line by the AY.4.2 S protein (Fig. 1f).

Monoclonal antibodies constitute an important treatment option for COVID-19, as they have been shown to reduce the risk of COVID-19-related hospitalization and death [9]. We tested whether AY.4.2 could be efficiently neutralized by five different clinically used antibodies that target the RBD. Four antibodies (casirivimab, imdevimab, etesevimab and sotrovimab) efficiently neutralized B.1, B.1.617.2 and AY.4.2 S proteins, while one antibody (bamlanivimab) was largely ineffective against B.1.617.2 and AY.4.2 (Fig. 1g), likely due to the L452R mutation [10] that is present in both S proteins.

With respect to neutralization by antibodies elicited upon infection or vaccination, we found no appreciable differences between the AY.4.2 and B.1.617.2 S proteins (Fig. 1h, i). Both S proteins were less efficiently neutralized by either convalescent plasma (median 1.6- and 1.3-fold reduction for B.1.617.2 and AY.4.2, respectively) or sera from BNT162b2/BNT162b2-vaccinated individuals (median 2.3- and 2.8-fold reduction for B.1.617.2 and AY.4.2, respectively) compared to the S protein of B.1 (Fig. 1h, i).

In summary, we did not observe appreciable differences in host cell entry or evasion of antibody-mediated neutralization between AY.4.2 and B.1.617.2. Thus, our data suggest that existing treatment options and vaccination will be equally effective against both variants.

## Supplementary information

Supplemental Material
