# Supplementary material for: No evidence for increased cell entry or antibody evasion by Delta sublineage AY.4.2
Source: Cell Mol Immunol. 2022 Jan 5;19(3):449–52. doi: 10.1038/s41423-021-00811-8 (PMC8727238; doi:10.1038/s41423-021-00811-8)
Supplement: Supplementary file 1 — Supplemental Material [file 41423_2021_811_MOESM1_ESM.pdf]

No evidence for increased cell entry and antibody evasion by Delta sublineage AY.4.2

Prerna Arora[1,2], Amy Kempf[1,2], Inga Nehlmeier[1], Luise Graichen[1,2], Martin S. Winkler[3], Martin Lier[3], Sebastian Schulz[4], Hans-Martin Jäck[4], Stefan Pöhlmann[1,2], Markus Hoffmann[1,2]

[1]Infection Biology Unit, German Primate Center, Kellnerweg 4, 37077 Göttingen, Germany

[2]Faculty of Biology and Psychology, Georg-August-University Göttingen, Wilhelmsplatz 1, 37073 Göttingen, Germany

[3]Department of Anesthesiology, University of Göttingen Medical Center, Göttingen, Georg-August University of Göttingen, Robert-Koch-Straße 40, 37075 Göttingen, Germany

[4]Division of Molecular Immunology, Department of Internal Medicine 3, Friedrich-Alexander University of Erlangen-Nürnberg, Glückstraße 6, 91054 Erlangen, Germany

Correspondence: Markus Hoffmann (mhoffmann@dpz.eu) or Stefan Pöhlmann (spoehlmann@dpz.eu)

Material and Methods

Cell culture

All cell lines were incubated at 37 °C in a humidified atmosphere containing 5% CO_2. 293T (human, female, kidney; ACC-635, DSMZ; RRID: CVCL_0063), Vero cells (African green monkey kidney, female, kidney; CRL-1586, ATCC; RRID: CVCL_0574, kindly provided by Andrea Maisner) and Huh-7 (human, male, liver; JCRB Cat# JCRB0403; RRID: CVCL 0336, kindly provided by Thomas Pietschmann) were cultured in Dulbecco's modified Eagle medium (DMEM, PAN-Biotech). Additionally, Calu-3 (human, male, lung; HTB-55, ATCC; RRID: CVCL_0609, kindly provided by Stephan Ludwig) and Caco-2 cells (human, male, colon; HTB-37, ATCC, RRID: CVCL_0025) were cultured in minimum essential medium (MEM, GIBCO). All media were supplemented with 10% fetal bovine serum (FBS, Biochrom), 100 U/ml penicillin and 0.1 mg/ml streptomycin (pen/strep) (PAN-Biotech). Caco-2 and Calu-3 cells were further supplemented with 1x non-essential amino acid solution (from 100x stock, PAA) and 1 mM sodium pyruvate (PAN-Biotech). Cell lines were validated by STR-typing, amplification and sequencing of a fragment of the cytochrome c oxidase gene, microscopic examination and/or according to their growth characteristics. Furthermore, all cell lines were routinely tested for mycoplasma contamination.

Expression plasmids

Plasmids pCAGGS-DsRed (1), pCAGGS-VSV-G (vesicular stomatitis virus glycoprotein) (2), pCG1- SARS-CoV-2 B.1 SΔ18 (codon-optimized, C-terminal truncation of 18 amino acid residues,

GISAID Accession ID: EPI_ISL_425259) (3) and pCG1-SARS-CoV-2 B.1.617.2 SΔ18 (codon-optimized, C-terminal truncation of 18 amino acid residues, GISAID Accession ID: EPI_ISL_1921353) (4) have been previously described. For S protein detection in immunoblot analysis, we further used pCG1-based expression plasmids for the respective full-length S proteins equipped with a C-terminal HA-epitope tag. To generate the expression plasmids for SARS-CoV-2 AY.4.2 S (based on GISAID Accession ID: EPI_ISL_5363764), we inserted the respective mutations into the pCG1-SARS-CoV-2 B.1.617.2 SΔ18 and pCG1-SARS-CoV-2 B.1.617.2 S-HA vectors by overlap-extension PCR. The pCG1 expression plasmid was kindly provided by Roberto Cattaneo, Mayo Clinic College of Medicine, Rochester, MN, USA. The integrity of all PCR-amplified sequences was verified by sequence analyses performed by a commercial service provider (Microsynth SeqLab).

Sequence analysis and protein models

All S protein sequences and the underlying information (collection date, location) were obtained from the GISAID (global initiative on sharing all influenza data) database (https://www.gisaid.org/). Protein models were are based on a template in which the SARS-2 S sequence was modelled on PDB: 6XR8 (5) using the SWISS-MODEL online tool (https://swissmodel.expasy.org) and further processed using YASARA (http://www.yasara.org/index.html).

Immunoblot

To investigate S protein cleavage and particle incorporation, vesicular stomatitis virus (VSV)

pseudotypes bearing S proteins with a C-terminal HA-epitope tag were concentrated by high-speed centrifugation (13,300 rpm, 90 min, 4 °C) through a sucrose cushion (20% w/v sucrose in PBS) and subsequently lysed in 2x SDS-sample buffer (0.03 M Tris-HCl, 10% glycerol, 2% SDS, 5% beta-mercaptoethanol, 0.2% bromophenol blue, 1 mM EDTA) by incubation at 96 °C for 15 min. After SDS-PAGE, proteins were blotted onto nitrocellulose membranes (Hartenstein) and blocked for 30 min in PBS-T (PBS containing 0.5% Tween 20) containing 5% skim milk. After blocking, membranes were incubated with primary antibodies against the HA tag (1:1,000, mouse, Sigma-Aldrich) or VSV-M (1:1,000, mouse, Kerafast) overnight at 4 °C. Subsequently, membranes were incubated with horseradish peroxidase-conjugated anti-mouse secondary antibody (1:2,000, Dianova). All antibodies were diluted in PBS-T containing 5% skim milk and after each antibody incubation blots were washed three times for 10 min with PBS-T. Immunoblots were developed using a self-made chemiluminescence solution (0.1 M Tris-HCl [pH 8.6], 250 μg/ml luminol, 0.1 mg/ml para-hydroxycoumaric acid, 0.3% hydrogen peroxide) in combination with the ChemoCam imaging system and the ChemoStar Professional software (Intas Science Imaging Instruments). Quantification of protein bands was performed using the ImageJ software (version 1.53C, https://imagej.nih.gov/ij/). For the analysis of S protein incorporation into VSV particles, total S protein signals (uncleaved, S0 and cleaved, S2) were normalized against their respective VSV-M signals and the resulting values were further normalized against the B.1 S protein (set as 1). For quantification of S protein cleavage, total S protein signals (uncleaved, S0 and cleaved, S2) were set as 100% for each S protein and the respective portions of S0 and S2 were calculated.

Production of VSV pseudotypes

Viral particles pseudotyped with the SARS-CoV-2 S proteins were produced as described previously (6). In brief, 293T cells were transfected with plasmids encoding S protein or VSV-G, or empty plasmid (control) using the calcium phosphate method. At approximately 30 h posttransfection, cells were inoculated with VSV-G-transcomplemented VSV*ΔG(FLuc), a replication-deficient vesicular stomatitis virus (VSV) that lacks the genetic information for its own glycoprotein (VSV-G) and instead codes for two reporter proteins, enhanced green fluorescent protein (eGFP) and firefly luciferase (kindly provided by Gert Zimmer) (7). After 1 h of incubation, the inoculum was removed and cells were washed with phosphate-buffered saline (PBS). Thereafter, to neutralize residual input virus, all cells received DMEM medium containing anti-VSV-G antibody (culture supernatant from I1-hybridoma cells; ATCC no. CRL-2700) except for cells expressing VSV-G, which received medium without antibody. After an incubation period of 16-18 h, the culture supernatant was harvested, clarified from cellular debris by centrifugation at 4,000 x g for 10 min, aliquoted and stored at -80 °C until further use.

Transduction of target cells

For transduction, target cells seeded in 96-well plates were inoculated with equal volumes of pseudotypes and transduction efficiency was evaluated at 16-18 h post transduction by measuring luciferase activity in cell lysates. For this, cells were lysed in PBS containing 0.5% Triton X-100 (Carl Roth) for 30 min at room temperature. Subsequently, cell lysates were transferred into white 96-well plates and mixed with luciferase substrate (Beetle-Juice, PJK) before luminescence was

measured using a Hidex Sense plate luminometer (Hidex).

VSV pseudotype-based neutralization assay

Collection of convalescent plasma and vaccinee serum samples and corresponding patient information have been described before (3, 4, 8). All serum and plasma samples were heat-inactivated at 56 °C for 30 min and pre-screened for their ability to neutralize transduction of Vero cells by particles pseudotyped with SARS-CoV-2 B.1 S. Neutralization assays were conducted as described (4, 8). In brief, S protein bearing particles were pre-incubated for 30 min at 37 °C with different concentrations (2, 0.2, 0.02, 0.002, 0.0002, 0.00002 μg/ml) of SARS-CoV-2-specific monoclonal antibody (Casirivimab, Imdevimab, Bamlanivimab, Etesevimab, Sotrovimab) or an unrelated control antibody (hIgG). Particles incubated with medium alone served as control (= 0% inhibition). Alternatively, pseudotyped particles were pre-incubated with different dilutions of convalescent plasma (1:50, 1:200, 1:800, 1:3,200 and 1:12,800) or serum from individuals vaccinated twice with the BNT162b2/Comirnaty vaccine (1:25, 1:100, 1:400, 1:1,600 and 1:6,400). Following incubation, mixtures were inoculated onto Vero cells with particles incubated with medium alone serving as control (= 0% inhibition). Transduction efficiency was determined at 16-18 h postinoculation as described above.

Statistical analysis

Data were analyzed using Microsoft Excel (as part of the Microsoft Office software package, version 2019, Microsoft Corporation) and GraphPad Prism 8 version 8.4.3 (GraphPad Software).

Statistical significance was assessed by two-tailed Students t-test with Welch's correction (S protein cleavage and cell tropism) or by Kruskal-Wallis analysis with Dunn's multiple comparison test (neutralization assay). Only p-values 0.05 or lower were considered statistically significant ($p > 0.05$, not significant [ns]; $p \leq 0.05$, *; $p \leq 0.01$, **; $p \leq 0.001$, ***). Plasma/Serum dilutions that lead to a 50 % reduction of transduction efficiency (neutralizing titer 50, NT50), were calculated by a non-linear regression model.

Supplementary Figure 1

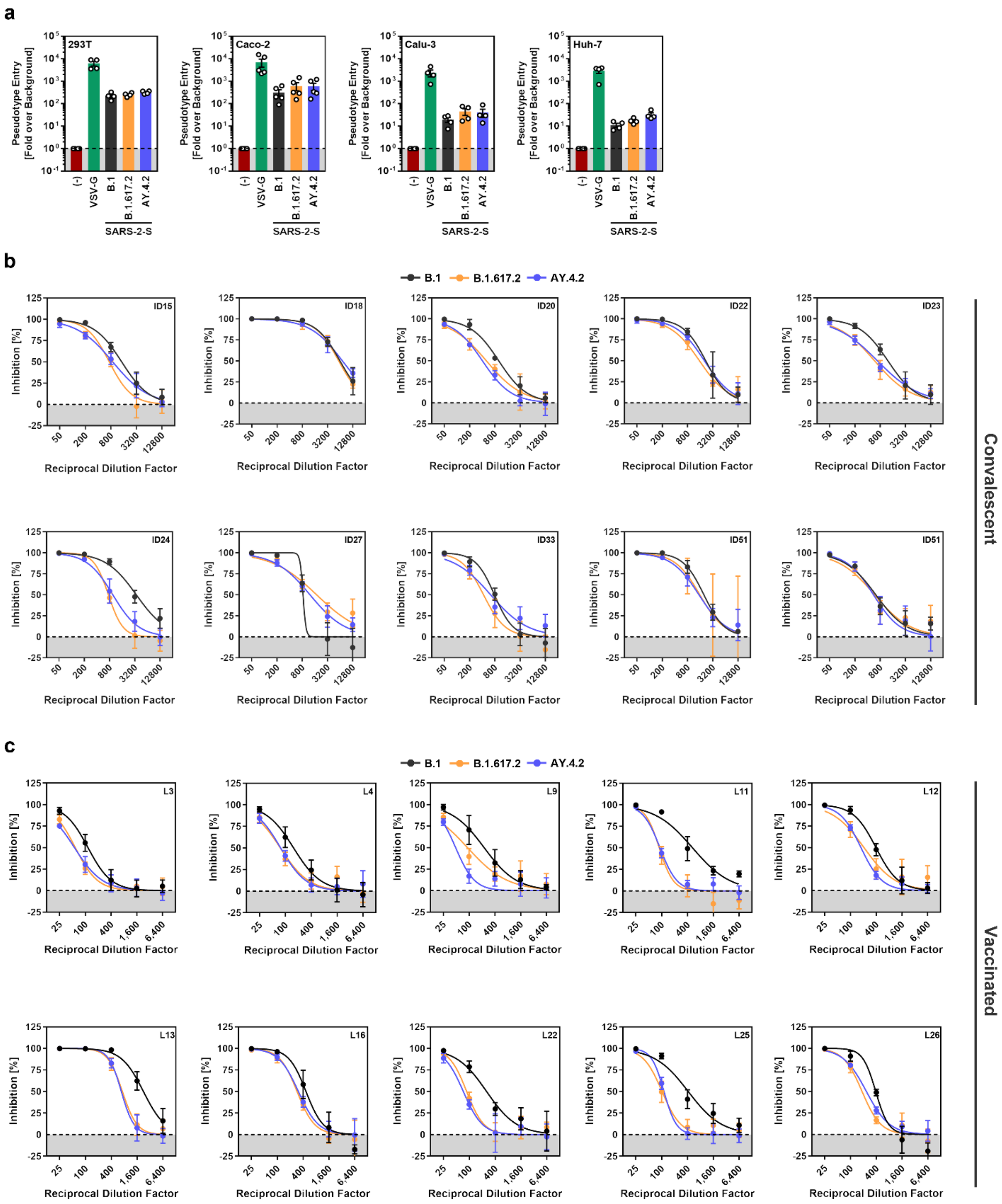

Cell tropism and neutralization of SARS-CoV-2 AY.4.2.

a Pseudotype entry data normalized against the assay background (related to Fig. 1f). The experimental procedure is outlined in the legend of Figure 1. The assay background is defined signals obtained from cell inoculated with particles harboring no viral glycoprotein (background, set as 1). Green bars further indicate pseudotype entry mediated by VSV-G. Error bars indicate the standard error of the mean.

b and **c** Individual neutralization data for each convalescent plasma (**b**) and vaccinee (BNT162b2/ BNT162b2) serum (**c**). Circles indicate mean values from four technical replicates and error bars indicate the standard deviation. Curves were calculated using a non-linear regression model (variable slope).

REFERENCES

1. Hoffmann M, Kleine-Weber H, Schroeder S, Kruger N, Herrler T, Erichsen S, et al. SARS-CoV-2 Cell Entry Depends on ACE2 and TMPRSS2 and Is Blocked by a Clinically Proven Protease Inhibitor. Cell. 2020 Apr 16;181(2):271-80 e8. PubMed PMID: 32142651. Pubmed Central PMCID: PMC7102627. Epub 2020/03/07.

2. Brinkmann C, Hoffmann M, Lubke A, Nehlmeier I, Kramer-Kuhl A, Winkler M, et al. The glycoprotein of vesicular stomatitis virus promotes release of virus-like particles from tetherin-positive cells. PloS one. 2017;12(12):e0189073. PubMed PMID: 29216247. Pubmed Central PMCID: 5720808.

3. Hoffmann M, Arora P, Gross R, Seidel A, Hornich BF, Hahn AS, et al. SARS-CoV-2 variants B.1.351 and P.1 escape from neutralizing antibodies. Cell. 2021 Apr 29;184(9):2384-93 e12. PubMed PMID: 33794143. Pubmed Central PMCID: 7980144.

4. Arora P, Sidarovich A, Kruger N, Kempf A, Nehlmeier I, Graichen L, et al. B.1.617.2 enters and fuses lung cells with increased efficiency and evades antibodies induced by infection and vaccination. Cell reports. 2021 Oct 12;37(2):109825. PubMed PMID: 34614392. Pubmed Central PMCID: 8487035.

5. Cai Y, Zhang J, Xiao T, Peng H, Sterling SM, Walsh RM, Jr., et al. Distinct conformational states of SARS-CoV-2 spike protein. Science. 2020 Sep 25;369(6511):1586-92. PubMed PMID: 32694201. Pubmed Central PMCID: 7464562.

6. Kleine-Weber H, Elzayat MT, Wang L, Graham BS, Muller MA, Drosten C, et al. Mutations in the Spike Protein of Middle East Respiratory Syndrome Coronavirus Transmitted in Korea Increase Resistance to Antibody-Mediated Neutralization. Journal of virology. 2019 Jan 15;93(2). PubMed PMID: 30404801. Pubmed Central PMCID: 6321919.

7. Berger Rentsch M, Zimmer G. A vesicular stomatitis virus replicon-based bioassay for the rapid and sensitive determination of multi-species type I interferon. PloS one. 2011;6(10):e25858. PubMed PMID: 21998709. Pubmed Central PMCID: 3187809.

8. Arora P, Rocha C, Kempf A, Nehlmeier I, Graichen L, Winkler MS, et al. The spike protein of SARS-CoV-2 variant A.30 is heavily mutated and evades vaccine-induced antibodies with high efficiency. Cell Mol Immunol. 2021 Oct 25. PubMed PMID: 34697413. Pubmed Central PMCID: PMC8543421. Epub 2021/10/27.
